# Relationships of Dietary Habits and Physical Activity Status with Non-Alcoholic Fatty Liver Disease Featuring Advanced Fibrosis

**DOI:** 10.3390/ijerph18178918

**Published:** 2021-08-25

**Authors:** Yoshito Yabe, Taeho Kim, Sechang Oh, Takashi Shida, Natsumi Oshida, Naoyuki Hasegawa, Kosuke Okada, Noriko Someya, Yuji Mizokami, Junichi Shoda

**Affiliations:** 1 Medical Sciences, Faculty of Medicine, University of Tsukuba, Tsukuba 305-8575, Japan; yoshitoyabe@outlook.jp; 2 The Center for Sports Medicine and Health Sciences, Tsukuba University Hospital, Tsukuba 305-8576, Japan; taeho.kim.my@gmail.com (T.K.); suncoral7411@gmail.com (N.S.); 3 Division of Radiology, Faculty of Medicine, University of Tsukuba, Tsukuba 305-8575, Japan; ohsechang@md.tsukuba.ac.jp; 4 Medical Technology and Science, International University of Health and Welfare, Narita 286-8686, Japan; t-shida@iuhw.ac.jp; 5 Division of Laboratory Medicine, Tsukuba University Hospital, Tsukuba 305-8576, Japan; n.u.al.th1@gmail.com; 6 Division of Gastroenterology, Faculty of Medicine, University of Tsukuba, Tsukuba 305-8575, Japan; hasegawa.naoyuki.gp@u.tsukuba.ac.jp (N.H.); yuji-mizokami@md.tsukuba.ac.jp (Y.M.); 7 Tsukuba Preventive Medicine Research Center, University of Tsukuba Hospital, Tsukuba 305-8576, Japan; okadakosuke2000@yahoo.co.jp

**Keywords:** non-alcoholic fatty liver disease, liver fibrosis, dietary habits, physical activity, Fibroscan-AST score

## Abstract

(1) Aim: Hepatic fibrosis is a prognostic factor for disease progression in non-alcoholic fatty liver disease (NAFLD). We aimed to determine the relationships between diet, physical activity, and the progression of liver fibrosis. (2) Methods: The 349 participants were categorized by their FibroScan-aspartate aminotransferase score, and they completed a questionnaire regarding their diet and physical activity. (3) Results: There were 233 patients in the negative-on-screening group, 78 in the gray zone group, and 38 in the positive-on-screening group. The frequencies of consumption of soybeans and soybean products and of light-colored vegetables were lower in the positive group; whereas the frequencies of consumption of snack food and fried sweets, jelly and pudding, fried food, and butter, lard, and beef tallow were higher. The odds ratios for the fibrosis progression in patients who consumed fried food ≥4 times/week was 2.21. The positive group also showed lower physical activity level (PAL) and exercise (Ex, metabolic equivalents for tasks (METs)/hour/week). The patients who undertook Ex at >7.5 had an odds ratio of 0.21 for the fibrosis progression. (4) Conclusion: High consumption of fried food and low Ex are risk factors for the fibrosis progression in NAFLD.

## 1. Introduction

In recent years, the number of people with obesity has been increasing in Japan because of the westernization of dietary habits and a lack of exercise. Furthermore, the prevalence of liver abnormalities has been increasing, according to the results of the recent national survey of human health check-up data; and an increase in the prevalence of non-alcoholic fatty liver disease (NAFLD) is thought to be the major factor driving this [1].

NAFLD is a phenotype of the metabolic syndrome that manifests in the liver [2]. NAFLD can be classified histologically as non-alcoholic fatty liver, in which triglycerides accumulate in hepatocytes; and non-alcoholic steatohepatitis (NASH), in which inflammation and fibrosis also develop. NASH may progress to cirrhosis and hepatocellular carcinoma. Histologic findings that affect the prognosis of NASH include fibrosis, portal vein inflammation, and ballooning, with the severity of fibrosis being a particularly important prognostic factor [3].

Dietary habits have a substantial influence on the development of hepatic fibrosis in NAFLD. Previous studies have shown that saturated fatty acids and trans-fatty acids in fatty foods have adverse effects on the liver in NAFLD [4,5], and that fructose in fruit is associated with greater intestinal bacterial growth and intestinal permeability [6,7]. In contrast, soybean products have serum lipid-lowering, anti-obesity [8], and anti-inflammatory effects [9], which may ameliorate fatty liver. Furthermore, the soluble fiber present in vegetables improves insulin sensitivity and affects the intestinal microflora [10].

Physical activity also influences the development of liver fibrosis in NAFLD. This represents the sum of the activities of daily living, such as work, housework, commuting, and schooling; and exercise, such as participation in sports, which is planned and deliberate, or regular physical activity for the purpose of maintaining and improving physical fitness [11]. We have previously shown that moderately intense aerobic exercise significantly ameliorates hepatic fat accumulation in NAFLD [12] and that high-intensity aerobic exercise reduces hepatic fibrosis [13] in patients with obesity and NAFLD. Thus, physical activity that is associated with high energy expenditure may ameliorate aspects of the pathophysiology of NAFLD.

Liver biopsy is the gold-standard method for the definitive diagnosis of liver fibrosis [14]. However, the number of patients with NAFLD in Japan is thought to be approximately 20 million, and therefore it is not practical to perform liver biopsies in all the patients who are suspected of having NAFLD to determine the extent of their liver fibrosis. Instead, non-invasive diagnostic tools are required [14], and fibrosis prediction formulas, such as the NAFLD fibrosis score and the FIB-4 index, have been used as means of non-invasively diagnosing fibrosis [15].

The FibroScan-aspartate aminotransferase score (FAST score) is derived from a combination of the results of liver stiffness measurement (LSM); liver fat content (controlled attenuation parameter; CAP), measured using ultrasound elastography (FibroScan); and serum aspartate aminotransferase activity [16]. The FAST score has been reported to be useful to reduce the number of patients who are placed in the high-risk NASH group [17]; that is, those with a NAFLD activity score (NAS) of ≥4 points and a fibrosis stage on histology (F) of ≥F2 [16]. Therefore, the FAST score can be used to noninvasively assess the progression of liver fibrosis in patients with NAFLD. Recently, a validation study of the FAST score was conducted in Japan and the results confirmed the diagnostic accuracy of the FAST score in Japanese patients with NAFLD [18].

In this study, we conducted a questionnaire survey of diet and physical activity in patients with NAFLD. The participants were categorized according to the tertiles of the FAST score [16] and their frequency of food intake and physical activity were compared. In this way, we aimed to elucidate the relationships of dietary habits and physical activity status with NASH featuring advanced liver fibrosis.

## 2. Materials and Methods

### 2.1. Participants 

**Figure 1:**
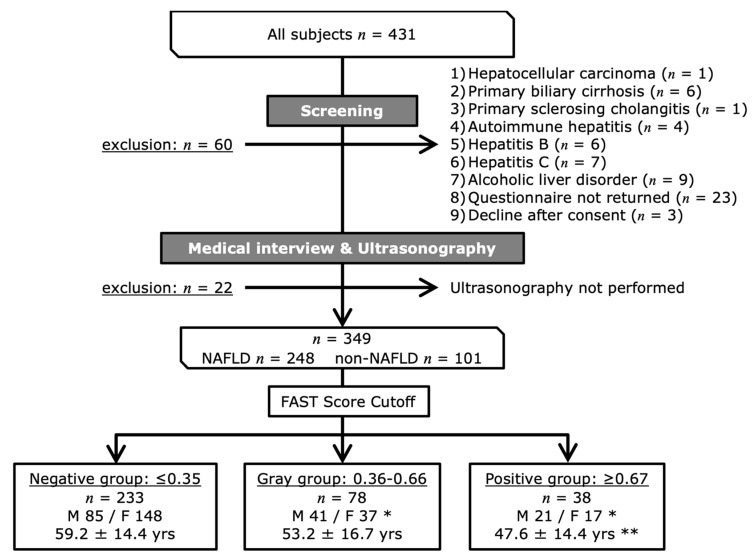
Flowchart of the enrollment and classification of the study participants. * The sex composition in the positive and gray groups was different from that in the negative group (*p* < 0.05). ** The mean age of the positive group was significantly younger than that of the negative group (*p* < 0.001).

The participants comprised 431 patients (184 men and 247 women; 57.1 ± 15.8 years old) who attended the outpatient clinic for lifestyle-related diseases at the Tsukuba University Hospital between January 2017 and March 2021 (Figure 1). They were referred to the hospital for the first time because they were found to have abnormalities such as obesity, liver dysfunction, dyslipidemia, glucose intolerance, and/or hypertension during a complete medical checkup or health screening. Patients who had a daily alcohol intake of >30 g/day for men and 20 g/day for women, who would be predisposed toward alcoholic liver disease, and those with other chronic liver diseases were excluded. Patients who declined to participate in the survey or who did not return the questionnaire and those who did not undergo examination of ultrasonography were also excluded (Figure 1).

Diagnoses of NAFLD were made by means of interview, ultrasonography, and ultrasound elastography. In total, 248 participants (118 men and 130 women; 55.8 ± 15.1 years old) were diagnosed as having NAFLD, and 101 (29 men and 72 women; 58.4 ± 18.2 years old) did not have NAFLD. The participants provided written informed consent to complete the questionnaire.

### 2.2. Questionnaire Survey Regarding Dietary Habits and Physical Activity 

We conducted a questionnaire survey of the diet and physical activity of the participants using the Food Frequency Questionnaire, based on food groups (FFQg, Ver.5.0) [19]. To analyze the data, Excel add-in software (Excel Eiyou-kun^®^, Kenpakusha Co. Ltd., Tokyo, Japan) was used. The questionnaire was administered by a researcher who verbally explained the response procedures and precautions to the participants, and the results were collected by mail.

In addition to the foods included in the five food categories of the Food Balance Guide (staple foods, main dishes, side dishes, milk and dairy products, and fruit) [20], we added foods that were shown to affect the pathogenesis of NAFLD in previous studies (Table 1). The participants were asked to choose their frequency of consumption of these foods from four options (never, 1–3 times/week, 4–6 times/week, or ≥7 times/week). The respondents were also asked to indicate the type and amount of alcohol that they drank each day, and the ethanol equivalents were calculated.

Physical activity level (PAL) and exercise (Ex) were calculated based on the responses to the physical activity questionnaire. PAL is an indicator of the intensity of average daily activities such as housework, commuting, and school work, and was calculated by dividing the energy expenditure calculated using the doubly-labeled water method by the basal metabolic rate of the participant. In adults, PAL is defined as I (low): 1.50, II (normal): 1.75, and III (high): 2.00 [21]. Ex is a unit that “expresses the amount of physical activity” by multiplying the intensity of the physical activity (metabolic equivalents for tasks; METs) per week by the duration of the physical activity (hours).

### 2.3. Anthropometric Measurements 

The measurement of body composition was analyzed by bioelectrical impedance analysis (BIA) [22]. The measurements were performed as described previously [23]. Obesity was defined as a body mass index (BMI) of ≥30 kg/m^2^. The following sarcopenia-related indices were calculated: SMI (limb skeletal muscle mass [kg]/height [m]^2^) [24]; SI (limb skeletal muscle mass [kg]/BMI) [25]; and SV ratio [26], an index of sarcopenic obesity. Grip strength and knee extension strength were measured as parameters of physical ability, as described previously [23].

### 2.4. Clinical and Laboratory Measurements

Most measurements were performed as described previously [23]. Parameters related to liver function, glucose metabolism, and lipid metabolism were measured as described in another previous report [12]. The homeostasis model assessment of insulin resistance (HOMA-IR) [27], NAFLD fibrosis score [28], and FIB-4 index [29] were calculated using the blood biochemistry data. Diabetes mellitus was diagnosed by a physician according to a fasting blood glucose level of ≥126 mg/dL, a blood glucose level after 2 h of ≥200 mg/dL OGTT, a casual blood glucose level of ≥200 mg/dL or HbA1c of ≥6.5%, on the basis of the criteria of the Japanese Society of Diabetes, and treatment with hypoglycemic agents was administered. Hypertension was defined as a physician-confirmed diagnosis and treatment with antihypertensive drugs, a systolic blood pressure of ≥140 mmHg, or a diastolic blood pressure of ≥90 mmHg, according to the Japanese Society of Hypertension criteria. Dyslipidemia was defined as either a physician-confirmed diagnosis and treatment with lipid-lowering drugs, or one of the following Japanese Atherosclerosis Society criteria: serum LDLC ≥ 140 mg/dL, triglycerides ≥ 150 mg/dL, or HDLC < 40 mg/dL.

### 2.5. Liver Stiffness and Steatosis 

Liver stiffness measurement (LSM) and controlled attenuation parameter (CAP) were determined by using a Fibroscan^®^ (Echosens, Paris, France) with a 3.5-MHz M probe for participants with BMI < 30 kg/m^2^ and with a 2.5-MHz XL probe for those with BMI ≥ 30 kg/m^2^. According to the validity criteria [30], the LSM reliability includes the following conditions: 10 valid acquisitions are available; the success rate is ≥60%; and the interquartile range to median ratio (IQR/M) is <30%. We decided to use the LSM data when all the above conditions were met. Measurements of LSM and CAP were performed by a single laboratory technician.

### 2.6. FibroScan-AST Score 

The FAST score [16] is a scoring system that is intended to non-invasively identify patients with NASH and significant active or progressive fibrosis. The FAST score was calculated using the LSM to assess liver fibrosis, CAP to assess liver fat mass, and AST, to assess liver inflammation. For the FAST score, the formula was used as follows: FAST score = e^−1.65+1.07×ln(LSM)+2.66×10−8×CAP3−63.3×AST−1^/1 + e^−1.65+1.07×ln(LSM)+2.66×10−8×CAP3−63.3×AST−1^ [16]. The participants were placed into three groups using their FAST scores (negative-on-screening, ≤0.35; gray zone, 0.36–0.66; and positive-on-screening, ≥0.67) [16].

### 2.7. Statistical Analysis 

Analyses were performed using SPSS version 26 (IBM. Inc., Armonk, NY, USA). Data are summarized as means ± standard deviations. The statistical significance level was set at 5%. To compare the basic characteristics and measured parameters among the groups, the Kruskal-Wallis and Mann-Whitney U-tests were used for continuous data, and the chi-square test for categorical data. The Dunn-Bonferroni test was used for multiple comparisons. The relationships of the FAST score with the frequency of food intake, PAL, and exercise level were analyzed using Spearman’s rank correlation coefficient. Furthermore, binomial logistic regression analysis was performed using models in which the progression of hepatic fibrosis (positive FAST score-on-screening, ≥0.67) was used as the outcome, and a frequency of food intake of ≥4 times/week, a PAL of ≥2.0, and an amount of exercise of ≥7.5 METs/hour/week were used as factors. The results are presented as odds ratios (ORs) with 95% confidence intervals (CIs).

## 3. Results

### 3.1. Categorization of the Participants According to Their FAST Score

The participants were placed into three groups using their FAST scores as follows: a negative-on-screening (negative) group, a gray zone (gray) group, and a positive-onscreening (positive) group (Figure 1). Comparisons of the basic characteristics of the three groups showed that the mean age of the positive group was significantly younger than that of the negative group (*p* < 0.001), and that the sex composition in the positive and gray groups was different from that in the negative group (*p* < 0.05).

### 3.2. Prevalences of Lifestyle-Related Diseases 

**Figure 2:**
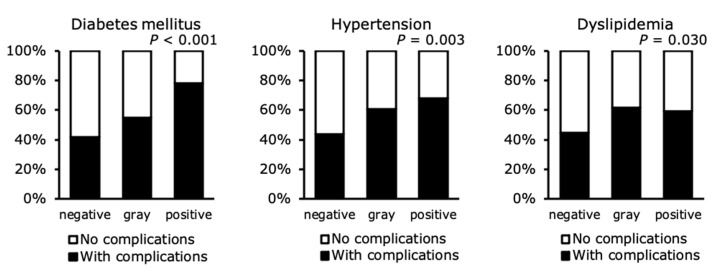
Prevalence of lifestyle-related diseases in participants placed in the negative, gray, and positive groups using their FAST scores. Data were analyzed using the chi-square test. FAST, FibroScan-AST; negative, negative-on-screening group; gray, gray zone group; positive, positive-on-screening group.

The prevalence of lifestyle-related diseases in the three groups was compared (Figure 2). The prevalence of diabetes mellitus significantly differed among the three groups and was highest in the positive group (gray vs. positive; *p* < 0.05, negative vs. positive; *p* < 0.01). The prevalence of hypertension also significantly differed among the three groups and was higher in the gray and positive groups than in the negative group (both *p* < 0.05). Finally, the prevalence of dyslipidemia significantly differed among the three groups and was higher in the gray group than in the negative group (*p* < 0.05).

### 3.3. Clinical Data 

The body mass, body composition, physical performance, blood biochemistry, liver fat content and elasticity, and liver fibrosis scores of the three groups are shown in Table 2.

### 3.4. Anthropometric Characteristics of the Groups 

The sarcopenia indices SMI and SI, and the sarcopenia-obesity index, SV ratio, were compared among the groups (Table 2). The SMI was significantly higher in the positive group than in the negative group. The SI was comparable in all three groups. The SV ratio was significantly lower in the positive group than in the negative group.

The waist-hip ratio and visceral fat cross-sectional area of the groups were also compared (Table 2). The waist-hip ratio was significantly higher in the positive group than in the negative group. The visceral fat cross-sectional area increased in proportion to BMI, and was significantly higher in the positive group than in the negative group.

### 3.5. NAFLD-Related Parameters among the Groups 

The serum AST, alanine aminotransferase (ALT), and gamma-glutamyltransferase (γGT) activities, which reflect liver dysfunction, were compared among the groups (Table 2). The activities of all the enzymes were higher in the gray and positive groups than in the negative group. Thus, liver dysfunction appeared to be worse in both the gray and positive groups than in the negative group.

All the parameters related to glucose metabolism increased in value with BMI. Specifically, fasting glucose, HbA1c, insulin, and HOMA-IR were higher in the gray and positive groups than in the negative group. For parameters related to lipid metabolism, serum HDLC concentration was significantly lower in the positive group than in the negative group, but the LDLC concentration did not significantly differ. The serum TG concentration was significantly higher in the positive group than in the negative group.

Transient elastography was used to evaluate liver fat content and fibrosis. CAP has been reported to reflect the fat content of the liver [31] and tended to increase in parallel with BMI and was higher in the positive group than in the negative group. LSM has been reported to reflect the extent of liver fibrosis [32], and LSM increased in parallel with BMI and was higher in the gray and positive groups than in the negative group (Table 2).

Liver fibrosis markers and liver fibrosis scores were also compared among the groups. Type IV collagen deposition was higher in the gray and positive groups than in the negative group and M2BPGi was significantly higher in the positive group than in the negative group. FIB-4 index did not significantly differ, although it tended to be higher in the positive group; and NFS also did not significantly differ, although it tended to be lower in the positive group.

### 3.6. Muscle-Related Characteristics 

Grip strength was measured as an index of muscle strength, and knee extension strength was measured as an index of muscle strength that reflects lower limb support, which is important for mobility and balance. Both parameters were higher in the positive group than in the negative group.

### 3.7. Biochemical Characteristics 

The levels of the inflammatory markers CRP and ferritin were compared among the groups. CRP was higher in the gray and positive groups than in the negative group, and ferritin was higher in the positive group than in the negative group.

### 3.8. Food Consumption Frequency 

**Figure 3:**
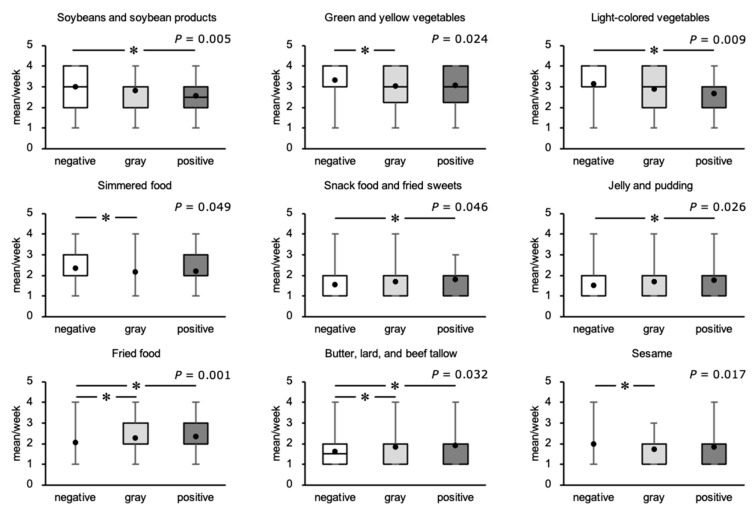
Frequency of consumption of food types by participants placed in the negative, gray, and positive groups using their FAST scores. Closed circles represent the mean values. Comparisons of the frequency of consumption of soybeans and soybean products, green and yellow vegetables, light-colored vegetables, snack foods and fried sweets, simmered food, jelly and pudding, fried food, butter, lard, and beef tallow, and sesame seeds per week among the three groups were made using the Kruskal-Wallis test and Dunn-Bonferroni test: * *p* < 0.05. negative, negative-on-screening group; gray, gray zone group; positive, positive-on-screening group.

We next compared the frequency of consumption of various foods by participants in the three groups (Figure 3). For soybeans and soybean products, green and yellow vegetables, light-colored vegetables, simmered foods, and sesame, the frequency of consumption differed among the three groups, with a lower frequency in the positive group or the gray group than in the negative group. For snack foods and fried sweets, jelly and pudding, fried foods, butter, and lard and beef tallow, the frequency of consumption differed among the three groups, with a higher frequency in the positive or gray groups than in the negative group.

We next analyzed the relationships between the FAST score and the frequency of consumption of the food types. The analysis showed significant correlations of the frequencies of consumption of soybeans and soybean products (ρ = −0.111, *p* = 0.038), green and yellow vegetables (ρ = −0.108, *p* = 0.043), light-colored vegetables (ρ = −0.124, *p* = 0.021), simmered food (ρ = −0.174, *p* = 0.001), snack foods and fried sweets (ρ = 0.151, *p* = 0.005), ice cream (ρ = 0.111, *p* = 0.038), jelly and pudding (ρ = 0.142, *p* = 0.008), fried food (ρ = 0.185, *p* = 0.001), sesame (ρ = −0.160, *p* = 0.003), salty snacks (ρ = 0.126, *p* = 0.019), and pickles (ρ = 0.114, *p* = 0.034) with the FAST score.

### 3.9. Physical Activity Status 

**Figure 4:**
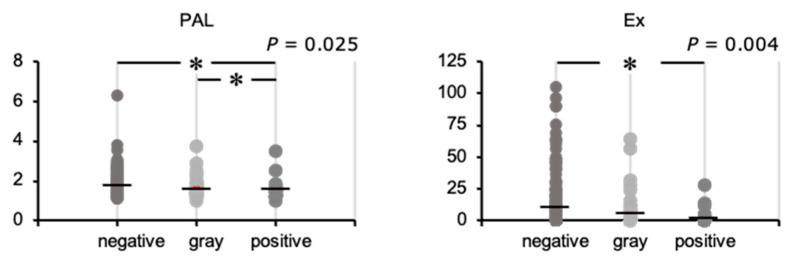
Physical activity level (PAL) and exercise (Ex) in participants placed in the negative, gray, and positive groups using their FAST scores. Bars represent the mean values. Comparisons of the physical activity level among the three groups were made using the Kruskal–Wallis test and the Dunn–Bonferroni test: * *p* < 0.05. negative, negative-on-screening group; gray, gray zone group; positive, positive-on-screening group.

We next compared the physical activity status of the three groups (Figure 4). There was a difference in PAL among the three groups; it was lower in the positive group than in the negative and gray groups. There was a difference in Ex among the three groups; this was lower in the positive group than in the negative group.

We analyzed the relationships of the FAST score with PAL and Ex performed. Correlation analysis showed that the FAST score significantly correlated with PAL (ρ = −0.123, *p* = 0.023) and Ex (ρ = −0.140, *p* = 0.009).

### 3.10. Relationship of Hepatic Fibrosis with the Frequency of Food-Type and Physical Activity Status

**Figure 5:**
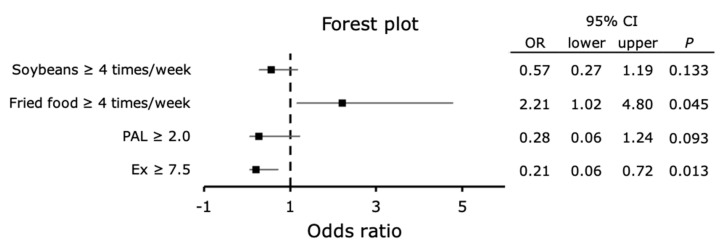
Relationships of dietary habits and physical activity status with the progression of liver fibrosis. The multivariable-adjusted relationships of the progression of liver fibrosis with the frequency of consumption of food items and physical activity status are shown. Outcome: FAST score screening positive: ≥ 0.67, Factor: frequency of food intake > 4 times a week, PAL ≥ 2.0, or Ex ≥ 7.5 METs/hour/week. The multivariable models included age, sex, PAL, Ex, and the frequencies of consumption of soybeans and soybean products, green and yellow vegetables, simmered food, snack foods and fried sweets, ice cream, jelly and pudding, fried food, sesame, salty snacks, pickles.

The relationship of the consumption frequency of food types with liver fibrosis was investigated using binomial logistic regression analysis (Figure 5). A multivariate model was created that included sex, age, foods correlated with the FAST score, and physical activity status, and a logistic regression analysis was performed with the outcome being a positive FAST score screening or higher. The significant items extracted were fried food ≥ 4 times a week (*p* = 0.045, odds ratio 2.21, 95% CI: 1.02–4.80) and Ex ≥ 7.5 (*p* = 0.013, odds ratio 0.21, 95% CI: 0.06–0.72).

## 4. Discussion

The prevention of the progression of NASH by lifestyle modification is an important issue in daily clinical practice. This study shows that patients with suspected NASH and advanced hepatic fibrosis, who were identified using the new FAST score, consumed soybeans and light-colored vegetables less frequently, and fried snacks and fried foods more frequently. Furthermore, they were found to have a low PAL and to do little Ex.

Hashidume et al. [8] reported that β-conglycinin, the major protein in soybeans, inhibits weight gain, reduces adipose tissue mass (an anti-obesity effect), and lowers blood glucose and liver triglyceride levels (a lipid-lowering effect). Soybean saponin has also been reported to have an anti-inflammatory effect [9]. In this study, we found that the frequency of consumption of soybeans and soybean products negatively correlated with the FAST score, and that the consumption of soybeans and soybean products ≥ 4 times/week was associated with less liver fibrosis (Figure 5).

According to the Food Composition Index [33], light-colored vegetables contain more water and dietary fiber than green-yellow vegetables. Dietary fiber can be classified as insoluble fiber and soluble fiber. Insoluble fiber is not easily fermented by intestinal bacteria, whereas soluble fiber is easily fermented [34]. The consumption of fermentable soluble dietary fiber has been shown to reduce body fat and ameliorate insulin resistance in patients with metabolic syndrome [10], via effects on the intestinal microflora [10]. In this study, the frequencies of consumption of green-yellow and light-colored vegetables negatively correlated with the FAST score.

The physiological effects of dietary fats differ according to the type of fatty acid they contain; therefore, it is important to focus on adjusting the frequency of intake of types of fatty acids, rather than to simply reduce total fat intake [35]. Specifically, saturated fatty acids induce lipotoxicity [4,36], and trans-fatty acids are involved in necrosis and apoptosis of hepatocytes in association with fat accumulation [5]. Saturated fatty acids are found in animal products, such as meat, butter, lard, beef tallow, and coconut oil. In contrast, monounsaturated fatty acids and n-3 polyunsaturated fatty acids reduce hepatic fat accumulation [37,38], and n-6 polyunsaturated fatty acids reduce hepatic lipid accumulation and improve metabolic status, without inducing weight loss [39].

Individuals who consume a large quantity of fried foods, snack foods, and/or fried sweets are likely to accumulate fat in their livers and experience fibrosis through the effects of the saturated fatty acids and trans-fatty acids contained in these foods on their intestinal tract and adipose tissue. This may explain the associations identified in this study. In contrast, there were no differences in the frequencies of the consumption of fish oil, perilla oil, egoma oil, and flaxseed oil, which contain high levels of n-3 polyunsaturated fatty acids; peanut oil, almond oil, olive oil, and canola oil, which contain high levels of monounsaturated fatty acids; or sesame oil, safflower oil, cottonseed oil, and soybean oil, which contain high levels of n-6 polyunsaturated fatty acids. This implies that variations in the consumption of these oils did not affect the progression of liver fibrosis in the participants.

It has been reported that exercise at ≥10 Ex reduces visceral fat mass [40] and liver fat [13]. However, few studies have determined the effects of physical activity on liver fibrosis in NAFLD. Exercise therapy is thought to slow the progression of hepatic fibrosis by improving glucose metabolism and ameliorating inflammation, through the maintenance of skeletal muscle function and a reduction in oxidative stress [41]. The findings of this study suggest that low activity levels and poor exercise habits may increase the risk of liver fibrosis in patients with NAFLD. Furthermore, they suggest that an exercise routine that involves ≥7.5 Ex, which is at the lower limit of the level recommended by the World Health Organization, may reduce the risk of progression of liver fibrosis.

The strength of this study is that it is one of the few that compares the results of surveys on food intake frequency and physical activity status, anthropometric data, and clinical laboratory data by classifying participants into three groups on the basis of the FAST score cutoff values: negative screening, gray, and positive screening. The novelty of this study is that in the screening-positive NASH group with advanced liver fibrosis, which was narrowed down using the new FAST score, a high frequency of fried food intake and even lower physical activity were found, which were speculated to be risk factors for the development of liver fibrosis.

This study had several limitations. In particular, first, we used the FFQ, a method of recording food intake frequency, as the dietary survey method. This approach has been reported to be associated with errors, owing to day-to-day variation [42] and under- and over-reporting [43]. Second, there were differences in the mean age and sex composition among the groups, which may have contributed to the differences in body composition, muscle strength, and the effect of sex hormones. Finally, of the various methods that can be used for the assessment of physical activity, we chose the use of a questionnaire method because of its ease of implementation. In this study, however, only 38 male and female participants with NASH and advanced liver fibrosis (in the positive-on-screening group) were enrolled. Therefore, to facilitate the statistical analysis, the data for men and women were analyzed together. In the future, we would like to study a larger number of participants with NASH and perform subanalyses according to age and sex.

## 5. Conclusions

A survey of diet and physical activity in patients with NAFLD showed that in those with suspected NASH and advanced hepatic fibrosis, frequent consumption of soybeans and soybean products was associated with less liver fibrosis, whereas the consumption of fried food was associated with more fibrosis. Furthermore, low PAL and Ex were associated with more hepatic fibrosis. Therefore, the dietary habits and physical activity status of patients should be considered and optimized as part of the management of NAFLD in daily clinical practice.

## Figures and Tables

**Table 1:** Questions comprising the Food Frequency Questionnaire.

(Q1)	Survey of daily physical activity	(Q25)	How many green and yellow vegetables do you eat?
1.	How many hours of sleep do you get?	(Q26)	How many light-colored vegetables do you eat?
2.	How many hours do you spend relaxing in a lying or sitting position?	(Q27)	How much oligosaccharide do you eat?
3.	How many hours of light work do you do sitting down?	(Q28)	How much jam and honey do you eat?
4.	How many hours of slow walking and housework do you do?	(Q29)	How much simmered food that uses sugar or mirin do you eat?
5.	How many hours of life activities and work are sustainable for a long time?	(Q30)	How many vinegared and dressed vegetables do you eat?
6.	How many hours of daily life activities/work do you need to rest frequently?	(Q31)	How many Japanese sweets do you eat?
7.	How many hours of exercise do you do every day, including weekends, excluding daily activities and work?	(Q32)	How much pastry and cake do you eat?
(Q2)	Survey of hours of exercise per week	(Q33)	How many snack foods and fried sweets do you eat?
1.	How many hours of exercise such as normal walking (less than 3 to 4 METs)?	(Q34)	How many rice crackers and cookies do you eat?
2.	How many hours of exercise such as fast walking (less than 4 to 6 METs)?	(Q35)	How much ice cream do you eat?
3.	How many hours of exercise such as jogging (less than 6 to 8 METs)?	(Q36)	How much chocolate do you eat?
4.	How many hours of running or other exercise (8–15 METs)?	(Q37)	How much candy and caramels do you eat?
(Q3)	How many people are in your family? Who is the main cook in your family?	(Q38)	How much jelly and pudding do you eat?
(Q4)	What is your current employment status?	(Q39)	How much preference beverage do you drink?
(Q5)	What is your current main occupation?	(Q40)	How much fried food do you eat?
(Q6)	What is your last educational background?	(Q41)	How much stir-fry do you eat?
(Q7)	What is the highest weight you have ever weighed?	(Q42)	How much mayonnaise and dressing do you use?
(Q8)	What was your weight when you were 20 years old?	(Q43)	How much margarine and fat spread do you use?
(Q9)	Are there any diseases you are currently suffering from?	(Q43)	How much butter, lard, and beef tallow do you use?
(Q10)	Have you had any illnesses in the past?	(Q44)	How many peanuts and almonds do you eat?
(Q11)	Are there any medications that you are currently taking?	(Q45)	How much sesame do you eat?
(Q12)	Do you eat three meals a day?	(Q46)	How much olive oil and canola oil (rapeseed oil) do you use?
(Q13)	What times do you eat meals each day?	(Q47)	How much safflower oil, cottonseed oil, and soybean oil do you use?
(Q14)	How much staple food do you eat?	(Q48)	How much perilla oil, egoma oil, and flaxseed oil do you use?
(Q15)	How many potatoes, pumpkin, and lotus root do you eat?	(Q49)	How many salty things do you eat?
(Q16)	How much fruit do you eat?	(Q50)	How many pickles do you eat?
(Q17)	How much meat and meat products do you eat?	(Q51)	How often do you use tabletop soy sauce and sauces?
(Q18)	How much fish and fish products do you eat?	(Q52)	How much miso soup do you eat?
(Q19)	How much seafood do you eat?	(Q53)	How much soup other than miso soup do you eat?
(Q20)	How many eggs do you eat?	(Q54)	How often do you eat noodles?
(Q21)	How many soybeans and soybean products do you eat?	(Q55)	How much of the noodle soup do you drink?
(Q22)	How much milk and milk products do you consume?	(Q56)	How do you feel about the taste of restaurants?
(Q23)	How much seaweed do you eat?	(Q57)	Do you currently smoke cigarettes?
(Q24)	How much small fish do you eat?	(Q58)	How much alcohol do you drink?

Modified from Yoshimura, Y. “Excel Eiyou-kun^®^ Ver.8.0 add-in software. Food Frequency Questionnaire New FFQg Ver.5.0”. Kenpakusha Co. Ltd., Tokyo, Japan (2016). Q; Question.

**Table 2:** Comparisons of clinical data among the groups.

		Negative			Gray			Positive			*p*	Multi-Comparison
		Mean		SD	Mean		SD	mean		SD		
BMI	kg/m^2^	24.6	±	4.3	29.6	±	6.1	34.5	±	9.5	<0.001	a
Skeletal muscle	kg	23.7	±	5.3	27.7	±	7.6	30.7	±	8.9	<0.001	a
Body fat percentage	%	30.6	±	9.3	35.8	±	7.6	39.9	±	8.6	<0.001	a
Waist-to-hip ratio		0.9	±	0.1	1.1	±	1.1	1.0	±	0.1	<0.001	a
SMI	kg/m^2^	6.8	±	1.0	7.7	±	1.5	8.4	±	1.7	<0.001	a
SI	kg/(kg/m^2^)	0.7	±	0.2	0.7	±	0.2	0.7	±	0.1	0.245	
SV ratio	kg/cm^2^	324.7	±	199.6	243.5	±	84.1	228.5	±	96.4	<0.001	a
Visceral fat cross section	cm^2^	91.7	±	42.0	125.6	±	47.1	151.9	±	61.4	<0.001	a
Knee Extension Stretch	kgf	36.8	±	12.3	42.9	±	16.0	45.9	±	16.9	<0.001	a
Grip (of hand)	kgf	28.9	±	8.3	32.5	±	10.3	32.4	±	9.8	0.005	a
ALB	g/dL	4.3	±	0.3	4.4	±	0.5	4.3	±	0.4	0.088	
AST	U/L	23.7	±	7.1	44.6	±	18.6	76.3	±	36.8	<0.001	a,b
ALT	U/L	24.3	±	14.7	68.0	±	43.0	103.9	±	51.8	<0.001	a,b
γGT	U/L	38.2	±	41.5	65.0	±	48.5	95.1	±	71.2	<0.001	a,b
HDLC	mg/dL	59.6	±	16.7	48.6	±	10.7	49.1	±	10.7	<0.001	a
LDLC	mg/dL	118.2	±	30.1	122.9	±	36.0	119.5	±	28.5	0.644	
TG	mg/dL	111.5	±	65.9	137.8	±	59.9	156.1	±	93.4	<0.001	a
Fasting glucose	mg/dL	110.8	±	27.2	117.8	±	30.2	131.9	±	38.0	<0.001	a,b
HbA1c	%	6.0	±	0.8	6.3	±	1.3	6.5	±	1.0	0.001	a,b
Insulin	μU/mg	9.9	±	11.7	19.4	±	18.1	25.8	±	14.6	<0.001	a,b
HOMA-IR		2.8	±	4.0	5.6	±	5.7	8.5	±	7.0	<0.001	a,b
Ferritin	ng/mL	96.8	±	77.9	164.4	±	196.0	213.2	±	294.0	0.001	a
Type IV collagen	ng/mL	111.0	±	29.5	131.9	±	49.8	188.0	±	117.5	<0.001	a,b
CRP	mg/dL	0.1	±	0.2	0.3	±	0.4	0.4	±	0.4	<0.001	a,b
M2BPGi	C.O.I.	0.7	±	0.4	1.2	±	1.9	1.3	±	1.1	<0.001	a
LSM	kPa	5.1	±	1.9	10.5	±	6.7	20.3	±	13.8	<0.001	a,b
CAP	dB/m	249.1	±	61.5	316.6	±	48.6	319.6	±	55.8	<0.001	a
FIB-4 index		1.6	±	2.2	1.7	±	1.5	2.2	±	1.8	0.394	
NFS		-1.6	±	1.6	−1.6	±	2.0	-1.0	±	1.9	0.05	

Values are presented as mean ± standard deviation. Comparisons among the three groups, categorized according to FAST score cut-off value, were made using the Kruskal-Wallis test. negative, negative-on-screening group; gray, gray zone group; positive, positive-on-screening group. a: *p* < 0.05 negative vs. positive, b: *p* < 0.05 gray vs. positive. FAST, FibroScan-AST score; BMI, body mass index; SMI, skeletal muscle mass index; SI, sarcopenic index; SV ratio, skeletal muscle mass-to-visceral fat area ratio; ALB, albumin; AST, aspartate transaminase; ALT, alanine aminotransferase; γGT, γ-glutamyltransferase; HDLC, high-density lipoprotein-cholesterol; LDLC, low-density lipoprotein-cholesterol; TG, triglyceride; HbA1c, hemoglobin A1c; HOMA-IR, homeostasis model assessment-insulin resistance; M2BPGi, Mac-2 binding protein glycosylation isomer; LSM, liver stiffness measurement; CAP, controlled attenuation parameter; NFS, NAFLD fibrosis score.

## Data Availability

The datasets analysed during the current study are available from the corresponding author on reasonable request.
